# Spatio-temporal impact of self-financed rotavirus vaccination on rotavirus and acute gastroenteritis hospitalisations in the Valencia region, Spain

**DOI:** 10.1186/s12879-020-05373-0

**Published:** 2020-09-07

**Authors:** Mónica López-Lacort, Alejandro Orrico-Sánchez, Miguel Ángel Martínez-Beneito, Cintia Muñoz-Quiles, Javier Díez-Domingo

**Affiliations:** 1 Vaccine Research Department FISABIO-Public Health, Avenida Cataluña, 21, 46020 Valencia, Spain; 2 Departament d’Estadística i Investigació Operativa. Universitat de Valencia, Valencia, Spain; 3 Unitat Mixta de Recerca en Mètodes Estadístics per a Dades Biomédiques i Sanitàries, Valencia, Spain

**Keywords:** Rotavirus, Vaccine impact, Spatio-temporal, Real-world data, Bayesian model

## Abstract

**Background:**

Several studies have shown a substantial impact of Rotavirus (RV) vaccination on the burden of RV and all-cause acute gastroenteritis (AGE). However, the results of most impact studies could be confused by a dynamic and complex space-time process. Therefore, there is a need to analyse the impact of RV vaccination on RV and AGE hospitalisations in a space-time framework to detect geographical-time patterns while avoiding the potential confusion caused by population inequalities in the impact estimations.

**Methods:**

A retrospective population-based study using real-world data from the Valencia Region was performed among children aged less than 3 years old in the period 2005–2016. A Bayesian spatio-temporal model was constructed to analyse RV and AGE hospitalisations and to estimate the vaccination impact measured in averted hospitalisations.

**Results:**

We found important spatio-temporal patterns in RV and AGE hospitalisations, RV vaccination coverage and in their associated adverted hospitalisations. Overall, ~ 1866 hospital admissions for RV were averted by RV vaccination during 2007–2016. Despite the low-medium vaccine coverage (~ 50%) in 2015–2016, relevant 36 and 20% reductions were estimated in RV and AGE hospitalisations respectively.

**Conclusions:**

The introduction of the RV vaccines has substantially reduced the number of RV hospitalisations, averting ~ 1866 admissions during 2007–2016 which were space and time dependent. This study improves the methodologies commonly used to estimate the RV vaccine impact and their interpretation.

## Background

Rotavirus (RV) is the leading cause of gastroenteritis in children < 5 years of age worldwide [1]. Prior to the license of the two live-attenuated rotavirus vaccines (RV1; Rotarix®, GSK and RV5; RotaTeq®, MSD) in 2006 and 2007, respectively, RV infection caused approximately 138 million episodes of acute gastroenteritis (AGE) per year (~ 2 million hospitalisations), of which ~ 3.6 million (~ 87,000 hospitalisations) occurred in Europe [2].

The World Health Organization (WHO) recommended including RV vaccination worldwide. The recommended schedule is two (RV1) or three (RV5) oral doses and should be completed between 6 and 32 weeks of age. Currently, 98 countries have introduced RV vaccines into their national immunisation programs [3]. This measure has had a major impact on the burden of AGE, decreasing RV outpatient visits and hospitalisations by 60–90% in Europe [4–7],

Although in Spain RV vaccines are recommended by the Spanish Paediatric Association but not funded by the National Health System (NHS), several post-authorization studies have also shown their effectiveness and impact on AGE and RV-AGE hospitalisations [8–12]. The Valencia Region of Spain could show a specific coverage-related impact of RV vaccines on AGE and RV-AGE hospitalisations and costs, despite the low-medium vaccine coverage (40–50%) [8].

Following WHO recommendations, most post-authorization studies usually estimate impact of the RV vaccine by comparing trends of RV or AGE hospitalisations in pre- and post- vaccination periods [7, 13, 14]. However, this ecological design is highly prone to bias and confounding [15–17].

In fact, a number of key studies have shown that the spread of infectious diseases are heterogeneously distributed in space because places differ in their environmental and population characteristics [18, 19]. Consequently, epidemiological studies are often confounded by complex and dynamic spatio-temporal processes [18, 20]. RV vaccine uptake and hospitalisations could, therefore, vary from time to time and between places for different reasons, including complex interaction of population demographics, socioeconomic inequalities, environmental factors, circulation of RV strains and their interactions across space and time [21]. Spatial variation in RV vaccination coverage [22] and in RV hospitalisations has been previously shown in the USA, Germany, Brazil, New Zealand [23–25].

A previous study in Spain showed strong variability in both vaccination coverage and RV/AGE hospitalisation rates over time and between health departments [8]. Thus, it would be important to evaluate variations in the RV/AGE hospitalisation risk and the impact of RV vaccination in a space-time framework to detect geographical-time patterns while avoiding the potential confusion caused by population inequalities in the impact estimates [7, 8, 12, 18, 24, 26].

Our aim is to assess the spatio-temporal impact of RV vaccines on RV and AGE-associated hospitalisations in children under 3 years of age in the Valencia Region using real-world data. In this study, real space-time rotavirus vaccination impact is predicted in terms of number of averted hospitalisations.

## Methods

### Setting and study population

This is a retrospective, population-based study using real-world data from the Valencia Region, including all children less than 3 years old living in the Region between 2005 and 2016.

The Valencia Region of Spain has approximately 4,900,000 inhabitants. Of them, around 3% (~ 150,000 children) are younger than 3 years old. The regional health system is divided into 34 public hospitals (24 of them with paediatric emergency rooms) and 241 health care districts structured into 24 health departments. As RV vaccines are administered to infants from six weeks of age, children with the first dose of RV vaccine recorded before six weeks of age were excluded from the study.

### Data sources

The Valencia Region has a set of multiple electronic databases collecting health and sociodemographic data from 98% of the population [27]. The population information system (SIP) was used to determine the population and their socio-demographics characteristics (sex, date of birth, health department, and health care district). Health care district and department are assigned by place of residence. Hospitalisations were collected from the minimum basic data set (MBDS). The vaccine information system (SIV) was used to obtain the vaccinated population; this source captures the immunisation history of each individual. Population, hospitalisation, and vaccination data were linked at individual level through a unique personal identification number [28].

### Outcomes and exposure

Our outcomes were identified from MBDS through a search of the following ICD-codes: (a) RV hospitalisations: hospitalisations with a discharge diagnosis of enteritis due to rotavirus (ICD-9-CM code 008.61, ICD-10 A08.0) in any diagnosis position. (b) AGE hospitalisations: hospitalisation with a discharge diagnosis of gastroenteritis-associated episode (ICD-9-CM codes 001–009, 558.9, 787.91; ICD-10 codes A00 – A09, K52.XX, R19.7) in any diagnosis position.

Vaccination status was assessed as a time-varying variable. Children were considered vaccinated from the date of the first dose of RV5 or RV1 and unvaccinated before that date. Children with no recorded rotavirus vaccination in SIV were considered as unvaccinated.

Vaccination coverage was calculated as the proportion of the children < 3 years old vaccinated with at least one dose of RV1 or RV5.

### Spatio-temporal analyses

The database for the analysis gathered population and hospitalisations aggregated by vaccination status, sex, age, health department, biennial periods (two-years period), and health care district.

A Bayesian spatio-temporal ecological model was constructed to analyse RV and AGE hospitalisation rates and to estimate the impact of vaccination on hospitalisations.

The model assumed that the number of hospitalisations (for RV or AGE) in the different observation units, *Y* = {*y*_1_, …, *y*_*vsadtm*_, …, *y*_*n*_}, followed a binomial distribution, where “v” indexes the two vaccination status, “s” the two sexes, “a” the 3 age groups (0, 1 and 2 years old), “d” the 24 health departments, “t” the 6 (biennial) periods, and “m” the 241 health districts. From now on, we will index *y* by *y*_*i*_ instead of *y*_*vsadtm*_ where i spans all the values of the sub-indexes v, s, a, d, t and m to make the notation shorter. Thus, the model assumed proceeds as follows:

$$ {y}_i\sim Bin\left({\theta}_i,{N}_i\right),i=1,\dots, 15,718 $$

Where *θ*_*i*_ is the hospitalisation rate and *N*_*i*_ the population for each observation unit. *θ*_*i*_ was modelled considering the logit link as follows:

$$ \log \left(\frac{\theta_i}{1-{\theta}_i}\right)=\log \left(\frac{\delta_m}{1-{\delta}_m}\right)+{\beta}_0+\sum \limits_{j=1}^3{\beta}_j{X}_j+{\alpha}_d+{u}_t+{v}_{tm} $$

where $\log \left(\frac{\delta_m}{1-{\delta}_m}\right)$ acts as an offset term to control for the hospital attraction (people who live near the hospital are more frequently admitted to it than those who live far from hospital, (see Additional file 1)), where *δ*_*m*_ is the estimated hospitalisation rate for all causes measured in each health care district. This rate was estimated using the spatial Besag-York-Mollié model [29] on hospital admissions for any cause. This offset makes that if no other term in the linear predictor had an effect, the corresponding risk, *θ*_*i*_, would be that corresponding to general hospital admissions for that health care district. *β*_0_ is the intercept term and *β*_*j*_ are the parameters associated with the categories of the covariates, *X*_*j*_: vaccination status, sex and age. The health department random effect, *α*_*d*_, was considered to fit the differences in admission policies between hospitals. *α*_*d*_ was considered to have the following distribution

$$ {\alpha}_d\sim N\left(0,{\sigma}^2\right), $$

where *σ* is also estimated within the model. No spatial dependence was considered for this term because it is expected to fit the admission policies of each hospital, which should not follow any spatial pattern. The biennial period effect, *u*_*t*_, was introduced to control the expected temporal variability in RV and AGE incidence. It was modelled as a random effect considering correlation between adjacent periods by a first order random walk modelled as an intrinsic conditional autoregressive (ICAR) prior distribution. Besides the temporal and spatial (health department) terms already mentioned, it was considered appropriate to include a spatio-temporal term that could jointly vary in time and space. The random effect *v*_*tm*_ reproduces this effect. This term is assumed to follow a spatio-temporal autoregressive model [30]. Thus, the spatio-temporal effect for the first period was formulated as

$$ {v}_{1m}={\left(1-{\rho}^2\right)}^{-1/2}{W}_{1m} $$

and for the following periods

$$ {v}_{tm}=\rho {v}_{t-1\;m}+{W}_{tm},t=2,\dots, 6, $$

where *W*_*tm*_ follows a spatial Besag, York and Molliè model [29] for each time period t inducing spatial dependence on *v*_*t m*_. On the other hand, *ρ* controls the temporal dependence in *v*_*t m*_. This parameter is assumed to follow a uniform prior distribution between − 1 and 1. Non-informative flat prior distributions were considered for *β*_*j*_ ( *j* = 0, . . , 3) parameters. Uniform prior distributions between 0 and 5 were considered for the standard deviations of all the random effects in the model.

Predictive distributions were used to estimate the number of rotavirus hospitalisations averted in order to assess the impact of rotavirus vaccination by health care district and time period. The number of cases averted by vaccination was calculated as the difference between the hospitalisations predicted by the adjusted model without the vaccine effect and the hospitalisations predicted by the model explained above.

R (Foundation for Statistical Computing, Vienna, Austria) and WinBUGS (Cambridge Biostatistics Unit and the Imperial College School of Medicine, London) software were used to perform the analysis using MCMC methods. A total of 2000 initial iterations were used as burn-in period of the MCMC. Subsequently, 10,000 iterations were run and only 1 in every 10 of them was saved. Three chains were simulated in total. MCMC convergence was assessed by visual inspection of history plots of posterior samples, the Brooks-Gelman-Rubin scale reduction factor, and the effective sample size implemented in the R2WinBUGS package of R. All statistical analyses conducted for this study are completely reproducible, and the data and the R code used for statistical analysis can be found as supplemental digital content to the paper.

## Results

The study included 721,471 children < 3 years old. Of these, 189,247 were vaccinated against RV. There were a total of 17,482 AGE hospitalisations, of which 28% (4871) were codified as RV. AGE and RV hospitalisations accounted for 8.4 and 2.4% respectively of all hospitalisations (207,014 hospitalisations for any cause). Vaccinated children accounted for 2248 AGE and 200 RV admissions.

### Spatio-temporal hospitalisation rate and relative risk

Risk of RV and AGE hospitalisations decreased with RV vaccination (Table 1). RV and AGE hospitalisation rates were 86% (95% CI: 84–88) and 47% (95% CI: 45–50) lower in vaccinees, respectively. Risk of RV and AGE hospitalisation also decreased with increasing age, by 72% (95% CI: 70–74) and 58% (95% CI: 56–60) respectively in two-year-old children as compared to those aged less than one year old. Risk of RV and AGE-hospitalisation was respectively 19% (95% CI: 15–23) and 15% (95% CI: 12–18) lower in girls as compared to boys. A strong variability in both RV and AGE hospitalisation rates was found between health departments (Additional file 2). Risk of AGE hospitalisation showed a downward trend during the study (Additional file 2), while the RV rate only declined between 2005 and 2010. Once controlled the vaccine effect, RV peaked in 2013–2014, with an 8% (95% CI: 6–14) higher rate than the average risk for the whole study period (Additional file 2). Additional structured spatio-temporal interaction was found for both outcomes. The spatio-temporal effect maps (Additional file 2) showed spatial clusters after adjusting for confounders.

**Table 1:** Model coefficients, Odds Ratio (OR) and 95% credibility interval (CI)

	RV		AGE	
	Coefficient, posterior mean (95% CI)	OR (95% CI)	Coefficient, posterior mean (95% CI)	OR (95% CI)
Intercept	−4.88(−5.01, − 4.76)		−3.78(− 3.88, − 3.67)	
**Vaccination Status**				
Unvaccinated	0	1	0	1
Vaccinated	−1.96(−2.11, − 1.81)	0.14(0.12,0.16)	−0.64(−0.68, − 0.59)	0.53 (0.5, 0.55)
**Age**				
0 years	0	1	0	1
1 year	−0.24(−0.3, − 0.18)	0.79 (0.74,0.84)	−0.16(− 0.19, − 0.13)	0.85 (0.82,0.88)
2 years	−1.28(− 1.36, − 1.2)	0.28 (0.26,0.3)	−0.87(− 0.91, − 0.83)	0.42 (0.4,0.44)
**Sex**				
Male	0	1	0	1
Female	−0.21(− 0.27, − 0.16)	0.81 (0.77,0.85)	−0.16(− 0.2, − 0.13)	0.85 (0.82,0.88)
Heterogeneity (random effect)				
Health department (unstructured)	0.28 (0.18,0.43)		0.22 (0.15,0.32)	
Health care district (unstructured)	0.08 (0,0.18)		0.05 (0,0.11)	
Health care district (structured)	0.38 (0.3,0.47)		0.32 (0.27,0.37)	
Period (structured)	0.19 (0.08,0.46)		0.17 (0.08,0.39)	
ρ	0.39 (0.15,0.6)		0.36 (0.21,0.5)	

See Additional file 2: OR and its 95% CI for period, health department, and spatio-temporal effects

### Spatio-temporal RV vaccination coverage

**Fig. 1:**
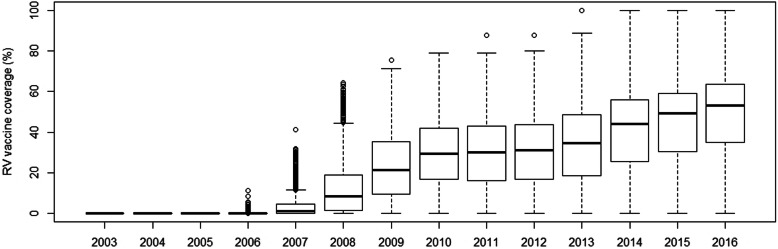
Description of RV vaccine coverage (%) by health care district and year

Rotavirus vaccination coverage varied considerably across the Valencia Region during the study period, with pockets of undervaccination (lower coverages) in many health care districts. Vaccination rates increased over the years in the districts. In 2016, 50% of the health care districts had a coverage higher than 53% (IQR: 35–64%) (Fig. 1). The overall RV vaccination coverage increased from 0 to 49% during the study period.

### Spatio-temporal RV vaccination impact

**Fig. 2:**
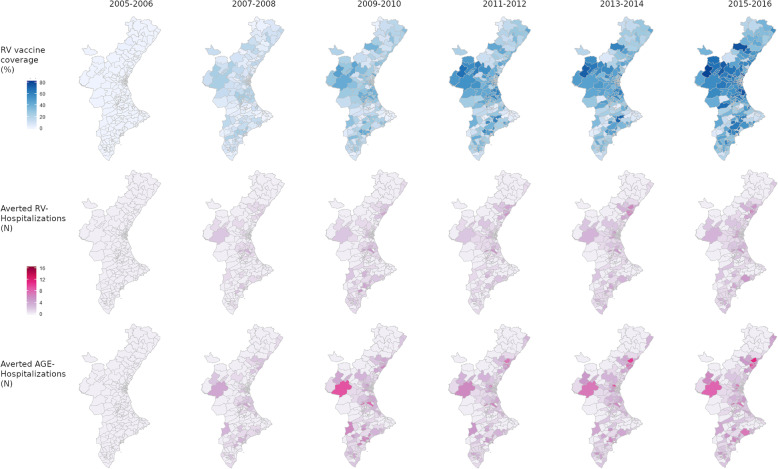
Spatio-temporal impact of RV vaccination on RV and AGE hospitalisations. *RV vaccine coverage (%) and number of averted hospitalisations by health care district and period estimated in the spatio-temporal model*

The number of hospitalisations averted by vaccination was coverage-dependent (Table 2), with impact of vaccination increasing as the number of vaccinees increased. With 189,247 children vaccinated, 1142 (95% CI: 1069–1222) RV and 1866 (95% CI: 1736–1992) AGE hospitalisations were averted. This represented overall reductions of 19.9% (95% CI: 19.7–20.2) in RV hospitalisations and 10.2% (95% CI: 9.7–10.5) in AGE hospitalisations for the whole period. The number of hospitalisations averted increased over time with increasing coverage. In 2015–2016, with a vaccination coverage of approximately 50%, there were reductions of 35.6% (95% CI: 35.2–36.1) and 19.7% (95% CI: 19.0–20.3) in RV and AGE hospitalisations respectively (Table 2). Maps in Fig. 2 show the distribution of RV and AGE hospitalisations averted by health care district over time. The impact on RV and AGE hospitalisations was greater in health care districts with higher coverage. Assuming 100% RV vaccine coverage, RV hospitalisations would be expected to be reduced by 85.8% (95% CI: 84.8–86.5) or 4920 (95% CI: 4602–5221) hospitalisations in the case of RV, and AGE hospitalisations by 46.9% (95% CI: 45.1–48.4) or 8606 (95% CI: 8056–9148) hospitalisations as compared to admissions if no child had been vaccinated during the study period.

**Table 2:** Impact of rotavirus vaccination on RV and AGE hospitalisations by period. Percentage and number of hospitalisations averted estimated by the model adjusted by age, sex, health care district, health department, biennial periods, and hospital attraction

				%, N (95% CI)	
Period	Children Vaccinated (N)	Unvaccinated (N)	RV Vaccine coverage (%)	RV Hospitalisations averted	AGE Hospitalisations averted
2005–2006	149	235,322	0.1	0%, 0 (0, 0)	0%, 1 (1, 1)
2007–2008	28,202	229,239	11.0	9%, 92 (84, 100)	5%, 169 (157, 180)
2009–2010	61,577	198,730	23.7	23%, 211 (193, 230)	13%, 390 (361, 420)
2011–2012	86,630	163,169	34.7	24%, 213 (193, 232)	13%, 359 (330, 387)
2013–2014	86,141	144,928	37.3	30%, 303 (274, 332)	16%, 446 (412, 482)
2015–2016	106,331	112,376	48.6	36%, 323 (295, 356)	20%, 502 (463, 543)

## Discussion

This is the first study estimating the spatio-temporal impact of RV vaccination on RV and AGE hospitalisations. The number of averted hospitalisations by RV vaccination was increasing in space and time in the Valencia Region during the study period in children < 3 years. Overall, ~ 1866 hospital admissions for AGE (potentially attributable to RV) were averted during 2007–2016. Despite the low-medium vaccine coverage (~ 50%) in 2015–2016, relevant 36 and 20% reductions were estimated in RV and AGE hospitalisations respectively. It should be noted that ~ 8606 hospitalisations would have possibly been averted during the whole study period if all children had been vaccinated. Direct benefits of vaccination were observed in the reduction of hospitalisation rates for RV (86%) and GEA (47%) in vaccinated children. These results are in accordance with the vaccine effectiveness estimated in the Valencia Region previously [9]. Regarding the spatio-temporal results, substantial variability was seen in RV vaccine coverage and hospitalisation risk for RV and AGE among health departments and health care districts. Spatio-temporal clusters were clearly distinguished. These patterns could be explained by climatic, environmental, sociodemographic, or economic differences, or by the different admission policies of health departments.

Although other impact studies reported relevant reductions in both RV and AGE hospitalisations in children < 5 years following RV vaccination [4, 6, 7, 13, 14, 31–33], only two of them showed a coverage-dependent response [8, 34]. Moreover, many of them were time-trend ecological studies comparing hospitalisation data in pre and post-vaccine populations and a historical pre-vaccine group [7, 13, 14, 33]. Even though this is the most commonly used method, it has been associated with potential confusion bias [15, 16]. The reported impact of the vaccination could be due to other secular trends caused by, changes in reporting, in medical practices, in health seeking behaviour, etc. [35]. Besides, vaccine impact based on hospitalisation data is prone to confounding, because hospitalisations rates are closely related to changes in the quality, access and use of the health care system which often occur simultaneously with introduction of new vaccines [17].

On the other hand, few spatial and spatio-temporal models have studied RV and AGE dynamics and none of them included the vaccination status of the population. Spatial variation in RV hospitalisations explained by sociodemographic characteristics of the population has previously been shown in studies conducted in Germany and New Zealand [23, 24]. Other studies in the USA and Brazil found that spatio-temporal variation in birth rate can lead to secular changes in the RV pattern [21, 25]. Finally, a study conducted in Bhutan showed that rainfall and temperature explain much of the spatio-temporal dynamics of diarrhoea (possibly due to RV infection in approximately 23% of cases) [31]. The studies developed in Germany and New Zealand were based in aggregated data over time, however, caution should be taken when interpreting this analysis because the area-specific risk may be overestimated or underestimated. Furthermore, none of these standard models considered spatio-temporal dependence; however, what occurs in a health care district is intimately related to what occurs in the adjacent one and is also related to what happened previously [36].

The present study analysed the impact of RV vaccination on RV and AGE hospitalisations from a different point of view. We developed a sophisticated spatio-temporal model that allowed us to estimate the RV vaccination impact in terms of adverted hospitalisations according to the number of children vaccinated. The spatio-temporal approach improves the commonly used methodologies to estimate the RV vaccine impact and its interpretation as follows. First of all, this analysis showed the evolution of the impact of RV vaccination and the risk of hospitalisation for RV/AGE in the Valencia Region at the health care district level over time. Second, adjusting by spatial variables such us health care district and health department in the analysis, several potentially attributable biases can be controlled. Those biases could have been caused by economic inequalities, environmental factors, socio-demographic differences or even possible changes in hospitalisations-admission policies [21, 37–39]. Moreover, the hospitalisation rate for any cause of each health care district was included to adjust the confusion caused by hospital attraction or other secular trends [17]. Finally, the Bayesian approach used allowed us to adequately capture dependencies among health areas and the potential relationship of data over time that cannot be easily modelled in classical statistics [40, 41].

Nevertheless, some limitations of our study should be highlighted. First of all, RV vaccines are not included in the official immunisation schedule, which may suggest differences between rotavirus vaccinees and non-vaccinees in terms of socioeconomic conditions and health-seeking behaviour. Therefore, socioeconomic factors might be an important confounder of our results and admissions at private hospitals should also be considered in future studies.

Secondly, although the positive predictive value of the rotavirus ICD-9-CM code identifying acute gastroenteritis attributable to rotavirus using MBDS resulted in 90% [9], different immunochromatographic methods with different sensitivities and specificities could have been used in the different hospitals during the study period [42]. In fact, based on the difference found in the number of hospitalisations prevented for AGE and RV (1866 vs. 1142), ~ 40% of underdiagnosis in RV hospitalisations was detected in the present study. Thirdly, health care district and health department could have varied over time; but only the last updated information was available. Fourthly, children who were unable to receive RV vaccines according to manufacturer recommendations (i.e. immunocompromised children) were not excluded from the analysis due to the lack of information.

Finally, it should be noted that both vaccines (RV1 and RV5) were used concurrently until 2010. But, RV5 was the only rotavirus vaccine available in Spain between 2010 and 2016. Therefore, results will have a limited value for estimating the impact of RV1.

## Conclusions

In summary, the introduction of the RV vaccines has substantially reduced the number of RV hospitalisations. The sophisticated spatio-temporal analysis allows us to show the impact of different vaccine coverage rates in terms of avoided hospitalisations in a geographical-time framework. Interestingly, our study predicted that ~ 8606 RV hospitalisations could have been adverted with all children vaccinated. This study improves the methodologies commonly used to estimate the RV vaccine impact and its interpretation. The spatio-temporal model avoided the potential confusion caused by population inequalities in the impact estimations. It also detects spatial clusters of the RV and AGE-hospitalisation risk attributable to common environmental, demographical, or cultural effects shared by neighboring regions.

## Supplementary information

**Additional file 1.**

**Additional file 2.**

## Data Availability

Additional analysis and results are available in RotApp AIV (rotapp.shinyapps.io/aiv2019). All statistical analyses conducted for this study are completely reproducible, and the data and the R code used for statistical analysis can be found in the following repository, https://drive.google.com/drive/folders/1UaZskXYjy7yYFSW7vpycTl_r4kcf6RwG?usp=sharing.

## Abbreviations

AGE: All-cause acute gastroenteritis
CMBD: Spanish hospital discharge database
CrI: Credible intervals
NHS: National Health System
OR: Odds Ratio
RV: Rotavirus
RV1: Rotarix® (GlaxoSmithKline Biologicals, Rixensart, Belgium)
RV5: RotaTeq® (Merck & Co., Inc., West Point, PA, USA)
SIP: Valencia’s administrative population-based database
SIV: Valencia’s Vaccine Information System
WHO: World Health Organization
