# Supplementary material for: Spatio-temporal impact of self-financed rotavirus vaccination on rotavirus and acute gastroenteritis hospitalisations in the Valencia region, Spain
Source: BMC Infect Dis. 2020 Sep 7;20:656. doi: 10.1186/s12879-020-05373-0 (PMC7487659; doi:10.1186/s12879-020-05373-0)

Additional file 2:

1.- OR and theirs 95% CI estimations for the period effect in comparison with the average of the whole period.


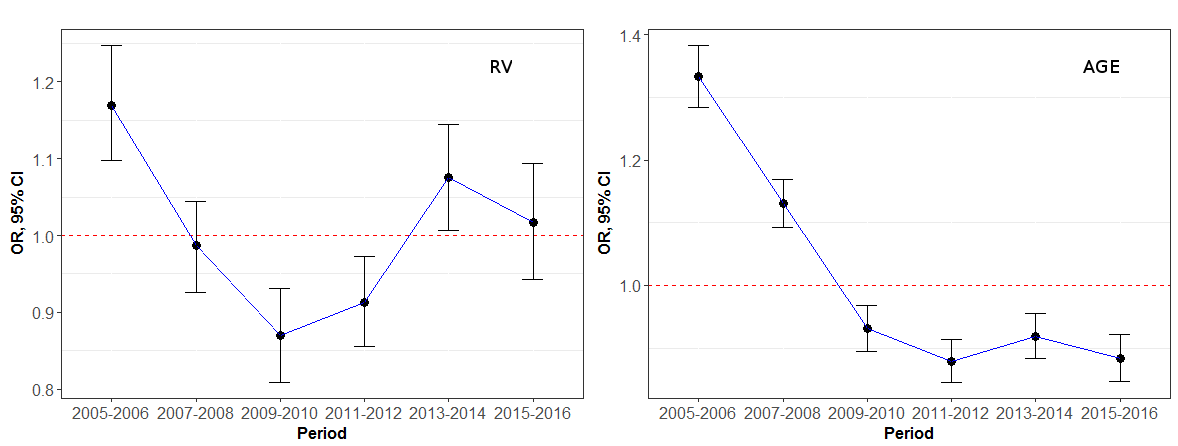


2.- OR and theirs 95% CI estimations for the health department effect in comparison with the average of all of them.


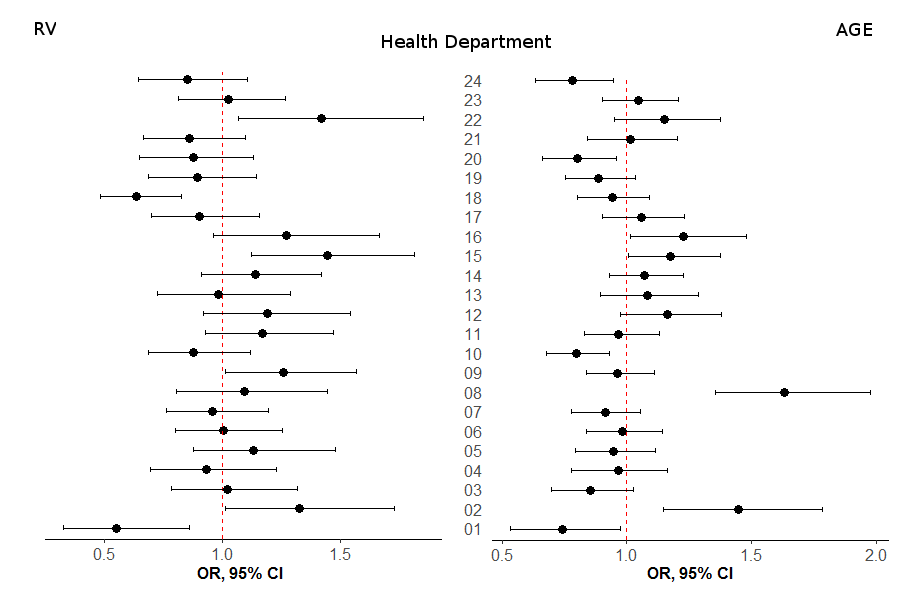


3.- Spatio-temporal risk of RV and AGE-Hospitalisation in comparison with the average of the Valencia R.
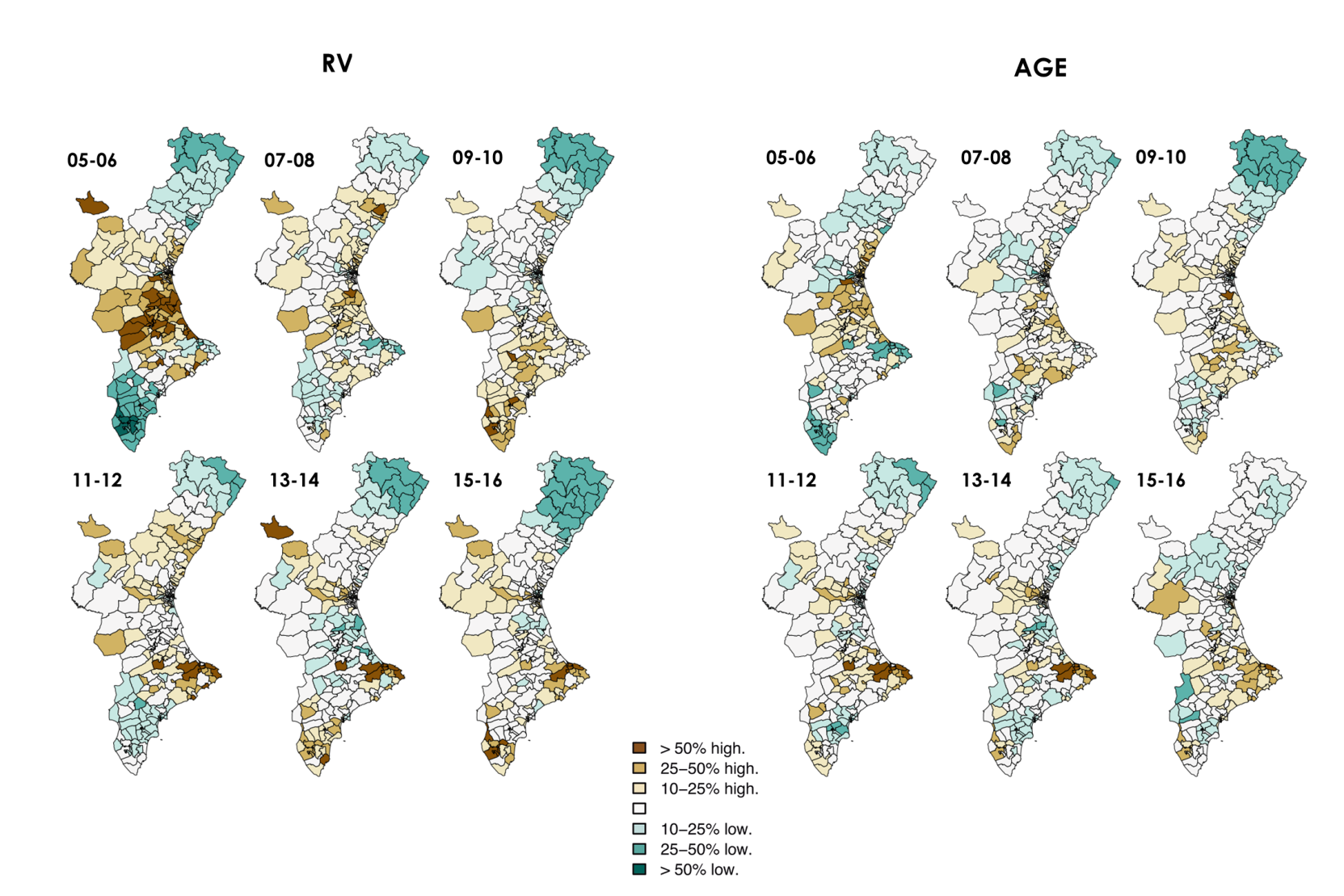

Supplement: Supplementary file 2 — Additional file 2. [file 12879_2020_5373_MOESM2_ESM.docx]
